# Anesthesia1President’s address delivered at the forty-first annual meeting of the Medical Association of Central New York, held at Buffalo, October 27, 1908.

**Published:** 1908-12

**Authors:** Carlton C. Frederick

**Affiliations:** Buffalo, N. Y.


### Anesthesia.[1]

By CARLTON C. FREDERICK. M. D., Buffalo, N. Y.

I WELL remember the trepidation when I first entered my hospital interne service with which I gave an anesthetic the first few times, for I knew nothing about doing it aright. It has always been the custom in most hospitals to place the latest ar-

1. President's address delivered at the forty-first annual meeting of the Medical Association of Central New York, held at Buffalo, October 27, 1908.

rival, the newest interne, in charge of the administration of the anesthetic during surgical operations and, in many instances, the same rule still obtains. There is no position of trust in the operating room, wherever it may be, that is so important to the patient except the operator himself. Yet, the anesthetic is given by any person who can hold a cone and can pour on the anesthetic, irrespective of his knowledge or experience in this very important part of the surgical proceedings. The dread that most patients have of the anesthetic, who are approaching an operation, is born either of former personal experience or from that of some friend. The old-fashioned paper cone, saturated with ether, thrust over the patient's mouth and nose, the suffocation and struggling during the first few moments of consciousness are certainly enough to make any one who has experienced it, or heard of it, dread the ordeal. Frankly, patients dread the anesthetic more than they do the operation in a large percentage of instances. Undoubtedly deaths following operation have more often been due to the anesthetic than the profession is aware of.

At this point I can do no better than quote from Dr. Baldy's address, at the last meeting of the American Gynecological Society, in which he gives a pen picture which any one of large surgical experience knows so well to be true. Who of you is not familiar with the terrible struggle for breath so common to the etherising room of the past, the congested, blackened face, the prolonged anesthesia, the patient only partly relaxed, the delay in the operation, the difficulties of the manipulation after an operation begun, the heartsickness at a difficult and delicate operation made double and trebly so from the unnecessary chances of sepsis, hemorrhage and shock, the feeling of a patient lost from no lack of skill of your own, the slipping of a ligature and a secondary operation or death, the immediate death on the table from failure of the heart, drowning due to inspired sputum, the vomiting on the operating table to the detriment of the operation, the prolonged after period of nausea and vomiting to the great suffering and misery of the patient, the inspiration pneumonias and other pulmonary complications, the nephritis and urinary suppressions, all due in great part to faulty anesthesia? How many deaths at the time of the operation, shortly after operation, or some days or weeks later are due to the same cause? What relation does the anesthetic bear to the large group of pulmonary complications reported from so many different sources, and what is its relation to the thrombosis and embolisms which have in the past caused so much suffering and disaster? What of the fatty degeneration of the liver, heart and kidneys? Who can tell?

It is neither the young or the old doctor who is asked to give the anesthetic who is to blame. They are qualified by reason of their degree, whether they are by experience or not. Why should a patient pay for the services of an experienced operator to rid him or her of the ills amenable to operation and, at the same time have the second most important part of the whole procedure relegated to a wholly inexperienced person. If they can afford to pay for the first, why should they not pay for the second, thereby securing as competent services from the anesthetist in his line as they do from the surgeon in his line?

Who then, is to blame for this condition of affairs? It is the medical profession in general, and the surgeons in particular. In other words, surgery has advanced and its handmaid, anesthesia has not. We have stuck to the old traditions, the old way of giving anesthetics, while surgery has made leaps and bounds in its attainments and possibilities. If the medical profession does not soon awake to the necessities involved and give more competent service in this line, an awakened public sentiment is going to lead to legislation which will force us to do so. Very few hospitals, especially the smaller ones and also some of the larger ones, have a paid anesthetist. They will have upon their wage list, superintendents, superintendent of nurses, housekeepers, laundresses, cooks, drivers, engineers, pharmacists, nurses, and the like, but no paid anesthetists.

The casual anesthetist is often forgetful of his patient and the anesthetic, in his zeal to watch the operation. The paid anesthetist usually has no leaning to surgical work, and it has little or no charm for him. The modern method of anesthetic administration, when done by one competent and experienced, is so little like that of old in its technic or results that one would hardly realise that there could be so great a difference.

I cannot here go into detail as to methods; that is not my purpose. I can only say that much of the dread of the first stages is needless today, as well as the subsequent nausea and distress. I have employed a paid anesthetist at the Buffalo Woman's Hospital for years and can truly say that at least 75 per cent. of my patients never vomit after operations, some of the remaining 25 per cent. have some nausea for the first few hours and about 10 per cent. vomit once, or possibly a few times. The patient is quickly brought under anesthesia and held quietly with no straining, no stertorous breathing, no vomiting to impede the operation, with perfect relaxation, yet with no mucous rales in the throat, or livid skin from congested capillaries. The old method of anesthesia has no place in the medicine of today and it devolves upon us to place it where it belongs among the relics of the past.

How? You who have connections with hospitals can insist on having some one person installed who knows or who will learn how, as official anesthetist and see to it that he or she is liberally paid for the service, for no competent person can afford to give their time in such service for a pittance. It has become quite the custom in some institutions to have a woman as an official anesthetist. Women make as good anesthetists as one can ask for. Their mental traits doubly fit them for this work, and in hospitals, there is always among the nursing staff one who can be trained to do the work satisfactorily. Some of the largest clinics in this country employ women in this capacity who have no other medical training. It requires knowledge of the essentials of the art first, then that to be supplemented by experience. The ablest medical man in the community may be the poorest anesthetist simply because of want of attention to its details and absence of practice.

What shall I say as to those parts of the country where there are no hospitals, where members of the community too seriously ill to be moved to a hospital must be operated upon in their homes. Most of us who operate have had plenty of these experiences when surgical work must be done under the most unfavorable and trying surroundings. I, myself, have seen times when operating, perhaps, at midnight in a farm house kitchen with smoky oil lamps for my only light and an untrained nurse as my only assistant, that I would have given all my expected fee for an anesthetist who would keep the patient from straining at one time and, at another time, having him so near complete narcosis that I wondered if he would ever breath again. And yet I ask the attending physician who calls me if I shall bring my assistant and my anesthetist? he replies, "No, we have plenty of help; all you need."

Cannot the physicians in every town induce some one of their number to look up the subject, get some practical instruction and then have him give the anesthetic in all cases needing it in their region so that he may supplement his training with absolute practical experience? Isn't this practicable? It seems to me that it is if the medical men in any community will get together, banish jealousies and get down to the practical question of what is best for their common interests in this matter. These suggestions may not be the best solution of the problems involved, but, if they will only serve to ameliorate the present conditions my object in making them will have been attained.

64 Richmond Avenue.

